# Linking Complement C3 and B Cells in Nasal Polyposis

**DOI:** 10.1155/2020/4832189

**Published:** 2020-07-08

**Authors:** Ulrike Werner, Axel Künstner, Maren Drenckhan, Ralph Pries, Karl-Ludwig Bruchhage, Hauke S. Busch, Claus Bachert, Barbara Wollenberg

**Affiliations:** ^1^Department of Otorhinolaryngology, University Hospital Schleswig-Holstein, Campus Lübeck, Germany; ^2^Group of Medical Systems Biology, Lübeck Institute of Experimental Dermatology, University of Lübeck, Germany; ^3^Institute for Cardiogenetics, University of Lübeck, Germany; ^4^Upper Airway Research Laboratory, Department of Otorhinolaryngology, Ghent University Hospital, Belgium; ^5^Division of ENT Diseases, CLINTEC, Karolinska Institute, Stockholm, Sweden; ^6^Clinic for ENT/HNS, Technical University of Munich, Germany

## Abstract

Nasal polyposis often is characterized by a persistent inflammation of the sinonasal mucosa, disease recurrence after medical or surgical intervention, and asthma comorbidity. Dysregulated complement activation may contribute to immunologic alterations and disease. To date, there is only scattered knowledge on the source and regulation of the central complement factors in the pathogenesis of nasal polyps. Here, we aim to study complement signatures, especially the C3-C3aR axis, and focus on cellular sources and targets in nasal polyps. Expression of complement factors, including C3, C5, and the anaphylatoxin receptors, was analyzed in nasal polyp tissue samples, the corresponding inferior turbinates, and healthy controls using transcriptomic methods and protein measurements. Distinct patterns of complement expression were found in nasal polyps compared to controls, characterized by an increased C3 activation and an increase in C3aR-bearing cells. In contrast, no difference was shown for epithelial-dependent C3 production. Besides low intracellular C3-expression levels for lymphocytes in general, we could identify an enlarged B lymphocyte population in nasal polyps displaying high amounts of intracellular C3. Our data suggest a prominent role for the C3-C3aR-axis in nasal polyps and, for the first time, describe a B cell population containing high levels of intracellular C3, suggesting a new role of B cells in the maintenance of the inflammation by complement.

## 1. Introduction

Chronic rhinosinusitis (CRS) is characterized by inflammatory changes in the sinonasal mucosa persisting at least 12 weeks and affecting 10.9% of the European population [1]. Chronic rhinosinusitis with nasal polyps (CRSwNP) is classified as one of the main subgroups of CRS. Nasal polyps are mostly raising from the middle nasal meatus [2] and are histologically characterized by lack of collagen [3], loose connective tissue with edema, and coverage with commonly pseudostratified respiratory epithelium [4]. Most forms of CRSwNP in patients of the western population show a T helper 2 (Th2) polarization with an infiltration of different inflammatory cells such as lymphocytes and macrophages but essentially consisting of eosinophilic granulocytes [5, 6]. Several hypotheses have been made to unravel the development of nasal polyps including the consideration of environmental factors such as fungi, *Staphylococcus aureus* with biofilm formation, and other microbial pathogens, but also host-specific factors such as an immune barrier dysfunction and alterations in the eicosanoid pathway [7–13]. Nevertheless, the exact most likely multifactorial mechanisms describing the pathogenesis, inflammatory processes, as well as the cellular progression still remain elusive [14].

The complement system, as an important part of the innate immunity, plays an important role in maintaining the immune homeostasis. The system consists of fluid phase plasma proteins and membrane-bound molecules and is divided into three distinct pathways—the classical, lectin, and alternative pathway [15]. Once activated, the three pathways lead to the formation of C3-convertases accompanied by C3 processing into C3b, opsonizing pathogens, and C3a, modulating inflammatory cells. In the further outcome, C5 is cleaved releasing C5b, the initial part for building membrane-attack complexes (MAC) lysing target cells, and C5a, a strong inflammatory mediator [16–18]. As the complement system is wide-ranging, a strict and fine-tuned regulation is indispensable. Nasal polyposis has a large immunologic background and especially the innate immune system is a promising field in unraveling the undiscovered aspects of this disease. So far, complement expression was shown to be upregulated in CRS(wNP) patients [19–21]. Anaphylatoxins were increased in nasal secretions of CRSwNP patients without displaying effects on serum levels [22]. The detection of complement activation products in tissue samples was demonstrated for CRS without polyps [20], but even more for CRSwNP patients suggesting a role for the classical pathway [22, 23]. Tan et al. reviewed recently the role of B cells and antibodies in CRS proposing a B cell-mediated classical complement activation [24]. Thereby, the expansion of extrafollicular activated B cells producing antibasement membrane autoantibodies leads to complement activation and epithelial damage. Anaphylatoxins, especially C3a being able to recruit eosinophilic granulocytes, might possess an important immune regulatory function in CRSwNP. The origin of higher complement load and activation still remains to be determined, whereas the immunologic cellular infiltrate might play a role.

Therefore, we aimed to investigate the link between complement signatures in CRSwNP, epithelial cells, and tissue infiltrating lymphocytes, especially B cells, as a potential regulatory loop to drive the progression of inflammatory nasal polyposis.

## 2. Materials and Methods

### 2.1. Ethics Statement

All patients were treated surgically at the Department of Otorhinolaryngology, University Hospital Schleswig-Holstein, Campus Lübeck, and have given their written informed consent. The study was approved by the local ethics committee of the University of Lübeck (approval number 16-278) and conducted in accordance with the ethical principles for medical research formulated in the WMA Declaration of Helsinki.

### 2.2. Patient Specimens

We examined tissue samples from 39 patients with CRSwNP (mean age 50.9, 29 male and 10 female) who belong to the western population, had a history of sinus-related inflammation for more than three months and did not respond to conservative therapy (Table 1). The nasal polyp tissue and corresponding inferior turbinate tissue of the same patient were harvested during sinus surgery to study differences between the inflamed polypoid tissue and inflamed nonpolypoid tissue. Patients were skin tested to several allergenic substances using standardized extracts (Allergopharma Joachim Ganzer KG) prior to surgery and classification of CRSwNP was determined by histopathological examination. Before surgery, all patients had been free of steroid medication for at least four weeks.

Additionally, the inferior turbinate from twelve healthy patients who underwent septal surgery or a septo-rhino-plasty (mean age 30.2, 9 male and 3 female) were harvested as noninflamed healthy controls.

### 2.3. Microarray

For microarray analysis, frozen tissue samples (*n* = 8) were sent to Miltenyi Biotec (Bergisch Gladbach, Germany). The mRNA was isolated using standard mRNA extraction protocols (Trizol), while the quality was evaluated with the Agilent 2100 Bioanalyzer platform (Agilent Technologies). RNA reaching an RNA integrity number >6 was used [25]. Agilent Whole Human Genome Microarrays (4×44K) were performed following manufacturer's protocols. Microarray analysis was performed using R version 3.5.2 with *limma* version 3.38.3 and the Affymetrix Human Genome U95 Set annotation data (Bioconductor package *hgu95a.db* version 3.8). The heatmap of genes involved in the complement pathways was constructed using the heatmap.2 function as implemented in the *gplots* package (version 3.0.1).

### 2.4. Quantitative Real-Time PCR

The microarray results were confirmed by quantitative real-time PCR (qRT-PCR) using the samples of the investigated seven patients together with six additional patients (*n* = 13) and further extended with healthy controls (*n* = 12) where RNA was extracted using the RNeasy™ Plus Mini Kit (Qiagen) following manufacturer's instructions for animal tissue. The quantity and purity of the RNA was determined using Nanodrop™ 2000 Spectrophotometer (Peqlab Biotechnologies) measuring at 260 and 280 nm. The cDNA was synthesized from maximum 1 *μ*g total RNA with the RevertAid First Strand cDNA Synthesis Kit (Thermo Fisher Scientific Inc.). QRT-PCR reaction mixture consisted of 10 ng cDNA, 10 *μ*L TaqMan™ Gene Expression Mastermix (Life Technologies), and 1 *μ*L 20x TaqMan™ Gene Expression Assays (Life Technologies) (Table 2).

The reaction was performed as shown before [26]. All reactions were performed in triplicate and *β*-actin was used as a reference control. The *Δ*Ct value (difference in threshold cycles for target and *β*-actin) was used in the 2^-*Δ*Ct^ formula to illustrate differential expression in the analyzed tissue samples. Ct values exceeding 35 cycles were assumed negative and expression was set to zero.

### 2.5. Western Blot

For western blot analysis, about 100 mg frozen tissue from CRSwNP patients (*n* = 15) was shredded in lysis buffer consisting of RIPA buffer (1% igepal® CA-630/0.5% natrium deoxycholate/0.1% sodium dodecyl sulfate (SDS) in PBS) complemented with 30 *μ*g/mL aprotinin, 1% phosphatase-inhibitor cocktail 2, 0.57 mM phenylmethylsulfonyl fluoride, 1 *μ*g/mL pepstatin A, and 0.5 mg/mL leupeptin (all from Sigma-Aldrich). After incubation for one hour on ice and centrifugation, the protein amount was determined via Bradford measurement.

Protein extracts (30-60 *μ*g) were mixed with SDS sample buffer (0.25 mM Tris-HCl pH 6.8, 8% SDS, 40% Glycerol, 20% *β*-Mercaptoethanol, 0.004% bromophenol blue in distilled water), and incubated for 5 minutes at 95°C. Proteins were separated by SDS-polyacrylamide gel electrophoresis using 7.5% or 10% gels with the peqGold protein marker V (PeqLab, VWR) added and transferred to 0.2 *μ*m nitrocellulose membranes (Bio-Rad) by electro blotting using a Mini PROTEAN® Tetra Cell (Bio-Rad). The membranes were blocked for one hour at room temperature in TRIS-buffered saline-0.1% Tween 20 (TBS-T) containing 5% bovine serum albumin (BSA) or 5% milk powder. Primary antibodies (anti-C3 1 : 3000, anti-C3aR 1 : 3000, anti-C5L2 1 : 500 from Novus Biologicals; anti-C5aR1 1 : 400 from Proteintech, anti-C5 1 : 100 from Acris Antibodies, anti-C3(activated) 1 : 100 from Santa Cruz Biotechnology; anti-GAPDH and anti-*β*Tubulin 1 : 1000 from Cell Signaling) were diluted in 3% blocking solution and incubated over night at 4°C. After rinsing in TBS, membranes were incubated with a Horseradish Peroxidase linked secondary antibody (Sigma-Aldrich) diluted 1 : 50000 in 3% blocking buffer for one hour at room temperature. Protein bands were detected through incubation with Amersham™ ECL™ Prime Western Blotting Detection Reagent (GE Healthcare Life Sciences) and measurement of chemiluminescence with the Fusion FX7 (Vilber Lourmat). To control equal protein loading, membranes were washed and immunologically stained as described above for GAPDH or *β*-Tubulin. Quantitative analysis was performed using Quantity One® 1D Analysis software, after which a normalization of target protein to loading control followed.

### 2.6. Immunohistochemistry

For cryostat sectioning, several fresh tissue probes (*n* = 7) were resected, embedded, directly frozen using liquid nitrogen, and stored at -80°C. Nasal polyp or inferior turbinate tissues were cryosectioned at 6 to 8 *μ*m with Cryostat CM 3050S (Leica Microsystems). The sections were stained immunohistochemically using the labelled streptavidin-biotin (LSAB) method for complement C3 (1 : 100, Novus Biologicals) and C5a (1 : 11, Acris Antibodies). The staining procedure was conducted as it was shown before [26]. For the exclusion of unspecific binding, control sections were stained only with the polylink secondary antibody. If necessary, staining intensity was evaluated by two independent investigators and determined with a scoring system: negative (-), weak (+), moderate (++), and strong (+++).

### 2.7. Primary Single Cell Suspension

Approximately, 1 g of fresh nasal polyp or inferior turbinate tissue (*n* = 10 for NP and *n* = 4 for cIT) was kept overnight at 4°C in tissue storage solutions (Miltenyi Biotech). The next day, the tissue was shredded manually in Dulbeccos's phosphate-buffered saline (PBS, Life Technologies) and centrifuged for 5 minutes at 1200 g. The enzymatic digestion followed for 2 hours in 37°C shaking water bath using the multitissue dissociation kit 1 (Miltenyi Biotech). After passing 70 *μ*m and 40 *μ*m cell strainer (BD), the suspension was centrifuged for enzyme removal and cells were resuspended in PBS for further procedures.

### 2.8. Flow Cytometry

Cells from primary single cell suspension were washed twice with PBS and centrifuged. First, a live-dead stain was performed for 30 minutes using Zombie-NIR™ (BioLegend) providing red fluorescence (APC-Cy7 channel) of dead cells. Afterwards, cells were washed, blocked (BD), and stained with fluorescent-labelled primary antibodies against extracellular antigens (one panel with anti-CD3-PE from BD; anti-CD19-APC, anti-CD45-PerCP, anti-CD14-PE-Cy7, anti-CD56-BV421™, and anti-CD16-BV510™ from BioLegend to analyze lymphocytes; another panel with BioLegend antibodies anti-EpCAM-AlexaFlour®647, anti-CD45-PerCP together with anti-CD3-APC-Cy7, anti-CD19-APC-Cy7, anti-CD14-APC-Cy7 as a dump channel for analysis of epithelial cells) for 30 minutes in 2% BSA/PBS. For the exclusion of irrelevant cell subsets (CD3/CD19/CD14) during staining procedures for epithelial cells, cells were additionally incubated with according APC-Cy7 labelled antibodies (BioLegend). After washing with PBS, cells were fixed with RBC lysis/fixation solution (BioLegend) for 15 minutes. The incubation with primary antibodies targeting intracellular epitopes (anti-pan-Keratin-PE from Cell Signalling Technology; anti-C3-AlexaFlour®488 and corresponding isotype control from Abcam) was performed in perm/wash buffer (BD) for 30 minutes at room temperature after 10 minutes blocking with 5 *μ*L 2% BSA/PBS in perm/wash buffer (BD). After washing with perm/wash buffer (BD), cells were resuspended in PBS and measured directly with BD FACS Canto II (BD). Approximately, 150000 to 300000 cells were recorded for each measurement.

For extracellular C3 staining of lymphocytes, the procedure terminated after incubation of primary surface antibodies at this including also anti-C3 antibody. An Ig-matched isotype control was used for C3 together with FMO controls to confirm gating strategies.

The gating procedure was realized as follows: during the analysis of single living untreated cells, a population being PE dim positive was apparent and was excluded from further analysis in all probes. Following gating procedure, doublets and dead cells were excluded first. CD45+ cells were then further analyzed for lymphocyte investigation and divided into CD3+ T cells and CD19+ B cells, whereas CD3-/CD19- were examined for CD16 and CD56 positivity. Since no discrimination between CD56^dim^ and CD56^high^ was possible and no consistent CD16 positivity was examined, NK cells were defined as CD3-/CD19-/CD16-/CD56+. Epithelial CD45- cells were gated for epithelial markers EpCAM (epithelial cell adhesion molecule) and pan-Keratin before C3 examination.

For analysis and interpretations, the BD FACS Diva Software 5.0.3 was used..

### 2.9. Statistical Analysis

All data are reported as mean + standard error of the mean (SEM). The analysis was done using GraphPad® Prism version 5.0 software (GraphPad Software). Differences between nasal polyps and corresponding inferior turbinates of the same patients were analyzed using Wilcoxon matched-pairs signed rank test or Mann–Whitney *U* test in case of unequal quantities. Differences between CRSwNP tissue and inferior turbinates of healthy patients were calculated with the Mann–Whitney *U* test. *p* values <0.05 were considered statistically significant.

## 3. Results

### 3.1. Complement Genes Are Differentially Expressed in Nasal Polyps

**Figure 1:**
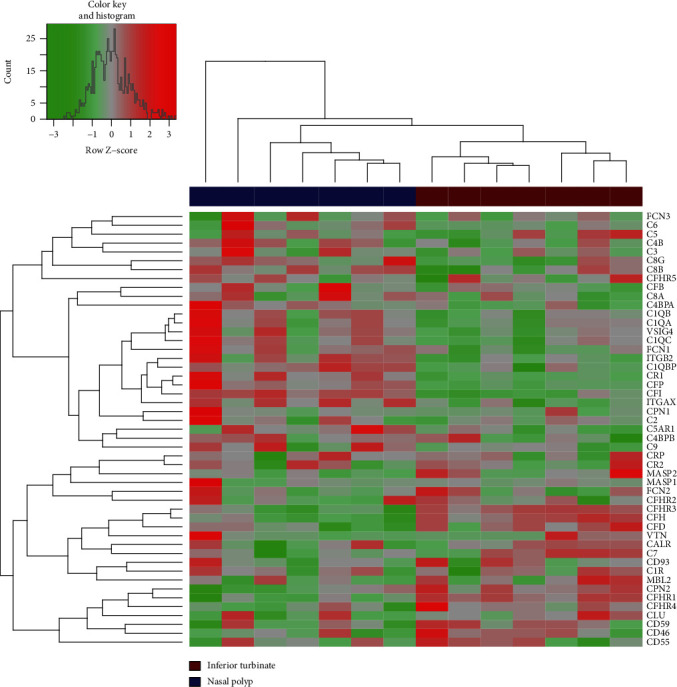
Heatmap of complement expression in nasal polyps compared to corresponding inferior turbinates after microarray analysis (*n* = 7).

Microarray analysis was performed using inflamed polypoid tissue and corresponding inflamed nonpolypoid tissue (inferior turbinate) of eight CRSwNP patients. One patient sample showing a greater discrepancy to the others was excluded (principle component analysis and inspection of the data; data not shown). The expression of several complement components is shown in the heatmap (Figure 1). Remarkably, several complement regulator genes (*CD59*, *CD46*, *CD55*, *CLUSTERIN*, *VITRONECTIN*, *CFH*, and *CFHR1-4*) were upregulated in inferior turbinates compared to nasal polyps, whereas some genes were increased in polyps, such as genes coding for complement receptors (*CR1*, *CR4*, *CRIG*); others only showed a slight increase for polyps as genes coding for the central cascade (*C3*, *C4*, *C5*, *C6*, *C8*). Also, properdin, the alternative pathway stabilizer, was highly abundant in nasal polyps. Notably, one nasal polyp sample seemed to be an outliner due to a more intense upregulation in many genes (leftmost sample in Figure 1).

These results show the important differences between nasal polyps and inferior turbinate illustrating a potential misdirected shift in complement expression in nasal polyps but also showing variances between the individual patients.

### 3.2. Central Complement Components Are Upregulated in CRSwNP Patients Compared to Healthy Controls

**Figure 2:**
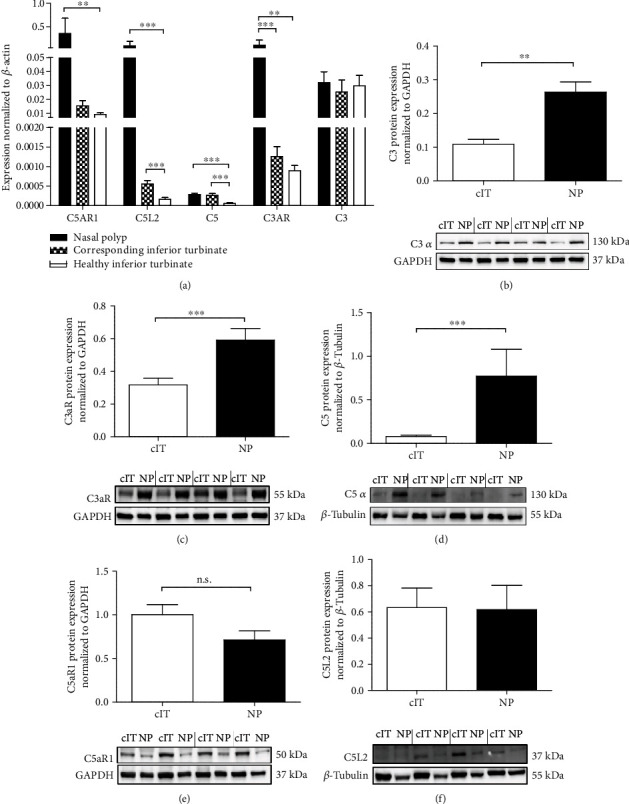
Complement C3, C5, and anaphylatoxin receptor expression in CRSwNP and healthy tissue. (a) The lysed tissue of nasal polyps (NP), corresponding (cIT), and healthy inferior turbinates was analyzed for mRNA expression of complement proteins by qRT-PCR displaying mean 2^-*Δ*Ct^ values. Significant differences were detected for C5AR1 (NP vs. hIT *p* = 0.0036), C5L2 (NP vs. hIT *p* = 0.0002/cIT vs. hIT *p* = 0.0007), C5 (NP vs. hIT *p* = 0.0004/cIT vs. hIT *p* = 0.0007), C3AR (NP vs. hIT *p* = 0.0039/NP vs. cIT *p* = 0.0007). *N* = 12 − 13. Means with SEM are shown. (b–f) Quantified protein expression of C3 (b, *p* = 0.0012), C3aR (c, *p* = <0.0001), C5 (d, *p* = 0.0009), C5aR1 (e), and C5L2 (f) comparing lysates of NP and cIT from CRSwNP patients (*n* = 15) in Western blots under reducing conditions with four representative patient samples. Means with SEM are shown. ∗*p* < 0.05; ∗*p* < 0.01; ∗∗∗*p* < 0.001.

Further extending the findings of microarray experiments, the complement components were tested in qRT-PCR experiments in the tissues from thirteen CRSwNP patients. No significant differences between nasal polyp (NP) tissue and corresponding inferior turbinate (cIT) could be measured for complement factor *C3*, *C5*, and the anaphylatoxin receptors for C5a (*C5AR1* and *C5L2*) (Figure 2(a)). In contrast, the anaphylatoxin receptor for C3a (*C3AR*, *p* = 0.0007) was significantly increased in NP compared to the cIT. The most striking difference can be seen between CRSwNP material compared to noninflamed control tissue of the inferior turbinate of twelve healthy donors, as most complement genes were significantly upregulated in nasal polyps (*C5p* = 0.0004, *C5AR1p* = 0.0036, *C5L2p* = 0.0002, *C3ARp* = 0.0039) or the corresponding inferior turbinate (*C5p* = 0.0007, *C5L2p* = 0.0007) versus controls, supporting the finding that the inflammatory reaction is also expanded to the inferior turbinate in patients with CRSwNP.

Characterizing the expression of C3, C5, and the anaphylatoxin receptors at protein level for 15 CRSwNP patients identified relevant end-point differences between the two types of tissues (NP vs. cIT, Figures 2(b)–2(f)). Our data demonstrate significantly increased expression levels of C3 (*p* = 0.0012), C3aR (*p* = < 0.0001), and C5 (*p* = 0.0009) in NP compared to the cIT (Figures 2(b)–2(d)). The receptors for C5a on the other hand were less abundant in polyp tissues (C5aR1, Figure 2(e)) or were not differently expressed between the polyps and the inferior turbinates (C5L2, Figure 2(f)).

### 3.3. Complement C3 Activation Is Enhanced in Polyp Tissue of CRSwNP Patients

Since the formation of C3b and C5a from native C3/C5 is an initiation pathway-independent parameter of complement activation, we analyzed C3b via Western blotting, and since C5a was not detectable with the same method it was analyzed via immunohistochemistry.

**Figure 3:**
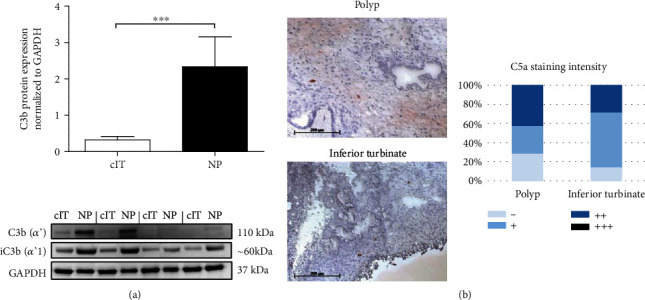
Complement C3 activation is higher in nasal polyps. (a) Quantified complement C3b and iC3b protein expression in lysates from nasal polyps (NP) compared to corresponding inferior turbinates (cIT) via reducing Western blotting (*n* = 15, *p* = 0.0004) with four representative patient samples. Means with SEM are shown. ∗∗∗*p* < 0.001. (b) LSAB-staining of C5a neo-antigen in polyp and corresponding inferior turbinate (*n* = 7). Representative images are shown (bar is 200 *μ*m). Staining distribution was scored as negative (-), weak (+), moderate (++), and strong (+++).

Significantly elevated levels of C3b were found in NP tissues compared to cIT (Figure 3(a), *p* = 0.0004) which is further cleaved to iC3b. The immunohistochemical staining intensity for C5a was evaluated using a scoring system [negative (-), weak (+), moderate (++), and strong (+++)] and revealed no apparent differences between NP and cIT (Figure 3(b)).

An increased C3 expression and activation was demonstrated in NP. Therefore, the subsequent step was to elucidate the cellular source of its expression.

### 3.4. Complement C3 Is Located at the Epithelial Compartment in CRSwNP Tissue

**Figure 4:**
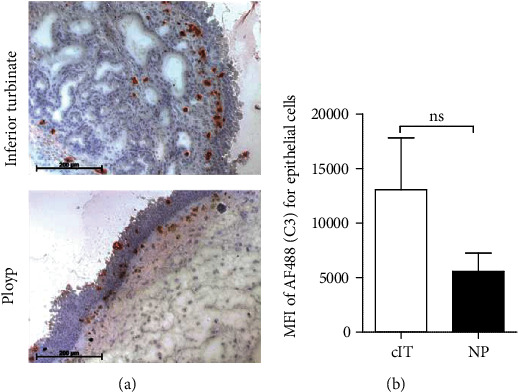
Complement C3 is located at epithelial cells in the nasal region. (a) The distribution of complement C3 was examined for nasal polyps and inferior turbinate with LSAB stainings. Example pictures are shown (bar is 200 *μ*m). (b) Using flow cytometry analysis, CD45-/EpCAM+/pan-Keratin+ cells were evaluated for MFI of AlexaFluor488® (C3, AF488) in a single cell suspension from primary CRSwNP patient tissues, corresponding inferior turbinates (*n* = 4) and nasal polyps (*n* = 9). Means with SEM are shown.

Although immunohistochemical C3 expression was partially shown in the lumen of cIT and NP tissue, the location of C3 was often near or in the epithelial compartment (Figure 4(a)). Therefore, epithelial cells were analyzed in NP and cIT single cell suspensions via intracellular flow cytometry staining to identify possible differences in the amount of C3. The AlexaFluor®488 (C3) mean fluorescence intensity (MFI) of CD45-/EpCAM+/pan-Keratin+ cells was evaluated and showed no significant difference between nasal polyps and associated inferior turbinates (Figure 4(b)).

### 3.5. A B Cell Subpopulation with Increased Intracellular C3 Is Found in Nasal Polyps

As potentially infiltrating immune cells could be a source for complement C3, single cell suspensions of nasal tissues were permeabilized for intracellular C3 analysis.

**Figure 5:**
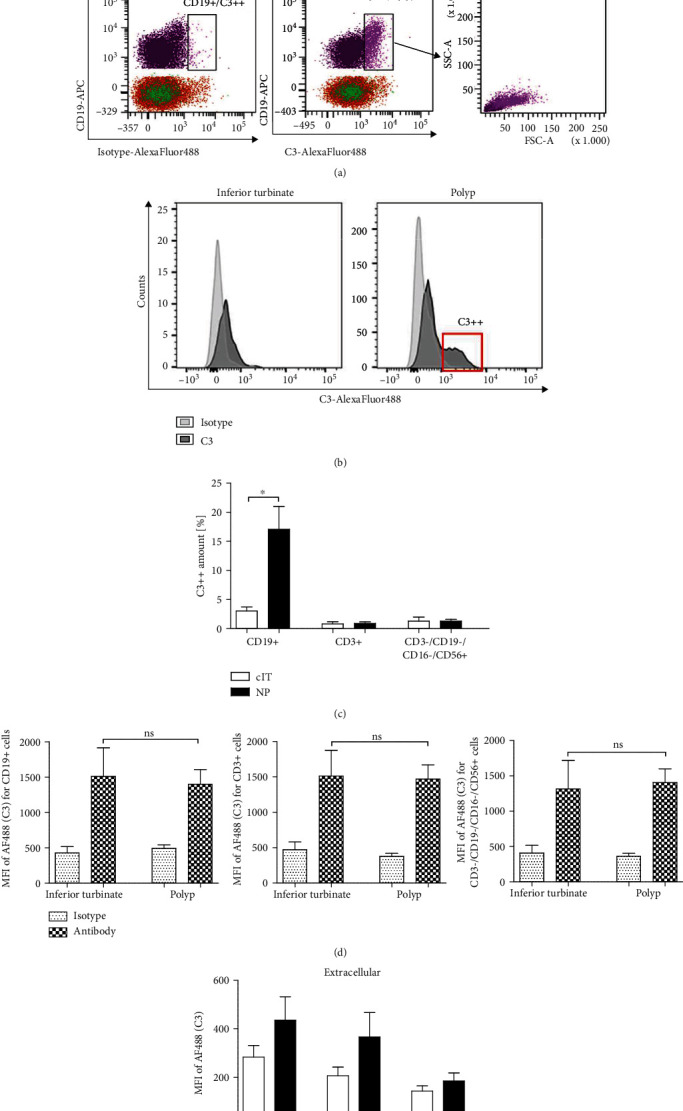
Larger B cells contain more intracellular C3 in nasal polyps. Polyp (*n* = 9) and inferior turbinate (*n* = 3 − 4) tissue samples were shredded and digested to prepare single cell suspensions. After excluding doublets and dead cells, lymphocytes were pregated for CD45. CD3+, CD19+, and CD3-/CD19-/CD16-/CD56+ immune cells were evaluated for C3 expression. (a) Gating strategy of nasal polyp with corresponding inferior turbinate. Immune cell subsets were gated by CD19-APC vs. C3-AlexaFluor488® compared to C3-matched isotype control. CD3+ (orange) and CD3-/CD19-/CD16-/CD56+ (green) cells are shown together in the lower part of the plots (CD19-), whereas CD19+ cells are displayed in violet or pink (high C3 expression: C3++), and the cellular size was further displayed using FSC and SSC. (b) Histogram of nasal polyp and corresponding inferior turbinate presenting C3-AlexaFlour488® signal for antibody (dark grey) and isotype control (light grey) of CD45+/CD19+ B lymphocytes with C3++ population (red box). (c) The high C3 positivity (C3++) is presented as percentage of immune cell subsets for nasal polyps (*n* = 9) compared to corresponding inferior turbinates (*n* = 3 − 4) (CD19+ cells *p* = 0.0196). Means with SEM are shown. ∗*p* < 0.05. (d) Intracellular C3 staining revealed a low C3-positivity for all cell types investigated in both tissues (NP *n* = 9, cIT *n* = 3 − 4) shown as MFI of AlexaFluor488® (AF488, C3) for isotype and antibody whereby C3++ CD19+ cells were excluded. Means with SEM are shown. (e) Extracellular staining of C3 without any permeabilization presented by displaying MFI of C3 and isotype control for lymphocyte subsets in nasal polyps (*N* = 5). Means with SEM are shown.

Flow cytometry results showed a low C3 expression (C3^low^) in all lymphocytes (CD3+, CD19+, and CD3-/CD19-/CD16-/CD56+) similarly in NP and cIT which is shown by displaying MFI for C3 and isotype control (Figure 5(d)). Surprisingly, a B cell population with higher intracellular C3 (C3++) was found in NPs (17.1 ± 3.9%, Figures 5(a) and 5(b)) and was significantly increased (*p* = 0.0196, Figure 5(c)) compared to cITs (3.0 ± 1.4%). Additionally, these B cells revealed an increase in cellular size compared to C3^low^ cells which is displayed in Figure 5(a) using forward and side scatter. No adequate C3++ population was thereby detected for other lymphocytes (Figures 5(a) and 5(c)). Since the used C3 antibody was raised against parts of the C3d amino acid sequence of C3 (Abcam), the possibility of surface bound C3 antibody on complement activated CD19+ B cells was ruled out via extracellular C3 staining without permeabilization. Comparing MFI of C3 and isotype control, no increased detection was shown on B cells which also corresponds to the other lymphocytes measured displaying similar behavior and therefore no relevant differences (Figure 5(e)).

## 4. Discussion

In this study, microarray analysis of tissues from nasal polyps and the neighboring lower turbinate revealed differences in complement gene expression pattern pointing to a potential misdirected shift to higher complement activation in nasal polyps.

Further, we concentrated on the detection of C3 and C5 as initiation pathway independent parameters. The formation of anaphylatoxins is of great importance for inflammatory responses, since they bind to their receptors (C3aR, C5aR1) by which immune cells are recruited and the release of inflammatory mediators is favored [16, 27, 28]. Van Zele et al. showed significant increased levels of complement C3a desArg and C5a desArg, the inactivated forms of C3a and C5a, in nasal secretions of CRSwNP patients compared to healthy donors [22]. In the context of different diseases such as atypical hemolytic uremic syndrome or paroxysmal nocturnal hemoglobinuria [29, 30], a dysregulated and persisting complement activity was already described as part of the inflammatory reactions. Our data of higher gene expression levels of anaphylatoxin receptors C3AR, C5AR1, and C5L2 in CRSwNP tissues, especially for nasal polyp tissues, compared to healthy controls is consistent with the higher load of immune cells expressing these receptors in the chronically inflamed surroundings.

In NP, an upregulated C3aR and C3 protein expression and activation was shown compared to cIT. In nasal polyps from individuals of the western population, the environment is dominated by a proinflammatory Th2-based immune response [31]. Especially the number of eosinophilic granulocytes is elevated in nasal polyps [6], whereas the recruitment of these cells via C3aR might be of great relevance. Drouin et al. demonstrated the importance of C3a-C3aR interaction for asthma and its Th2 response, whereas C3aR deficiency leads to a decrease in eosinophils and diminished Th2-related cytokines [32]. Also, Mulligan et al. published observations showing reduced inflammation during C3aR inhibition in an *Aspergillus fumigatus*-induced CRS mouse model [19], supporting the detected results and the importance of C3a-C3aR interaction in humans. Nevertheless, although we could not show a prominent role for C5a and associated C5aR-bearing cells in NP, others postulate a role for increased MAC-formation along the epithelium and blood vessels leading to damage and loss of function [22, 23]. Therefore, the role of C5 activation needs to be further determined.

The upregulated burden of complement components in nasal polyps raised the question of which cells are involved in the complement protein syntheses. Complement production has been described for several epithelial cells [19, 33] to be higher in nasal polyps [19]. We could demonstrate immunohistochemical C3 deposition at the epithelium and in the lumen in nasal polyps, but also for the inferior turbinate. Flow cytometric analyses of epithelial cells extracted from fresh primary tissue samples showed intracellular C3, whereas no differences could be displayed between NP and cIT. Therefore, although an increased complement activation might occur at epithelial areas [22, 23], the intracellular C3 amount is not enhanced in epithelial cells of nasal polyps.

For all lymphocytic subgroups analyzed, a low intracellular C3 expression (C3^low^) was detected in NP and cIT, coherent with previous findings for various immune cells (mast cells, monocytes, macrophages, dendritic cells, lymphocytes [34, 35]). For T-lymphocytes, intracellular complement C3 was shown to be important sustaining homeostasis [28]. C3 in blood-derived B cells was shown to be synthesized on a low level by the cell, but mostly being taken up from the surrounding [28, 36, 37].

Surprisingly, we could demonstrate a CD45+/CD19+ B cell population, making up about 17% of total B cells, with higher intracellular C3-expression (C3++) which is significantly increased in nasal polyps compared to inferior turbinates and could not be shown consistently for other immune subsets investigated. At the same time, increased FSC of the C3++ B cells compared to C3^low^ cells indicated an increased size and/or activation. That would propose that possibly activated B cells, becoming larger during activation and differentiation [38], contain detectable higher intracellular C3 levels in nasal polyps. This would be in accordance with elevated levels of factors playing a role in B cell proliferation, differentiation into plasma cells as well as antibody diversity shown in nasal polyps [39–42]. Even higher amounts of plasmablasts and plasma cells together with more antibodies and autoantibodies were found in nasal polyp tissue compared to controls [24, 39, 40, 43, 44]. Therefore, the detailed characteristics of the C3++ B cell subpopulation remain to be determined. Although the utilized C3 antibody was not detected on the B cell surface, the corresponding significantly more abundant split products might additionally lead to excessive B cell activation which could be examined using C3d-specific antibodies. Tan et al. recently reviewed the role of B cell activation and antibody formation in CRS proposing a B cell-mediated classical complement activation [24], which is supported by others suggesting complement activation via autoantibodies [22, 23]. In context with our results, linking activated B cells with a higher C3 load in nasal polys, this would support an idea of a dysregulated B cell-dependent C3 production together with its activation in nasal polyps which potentially might result in an interactive activation loop of B cells and complement. Following the reports of Kremlitzka et al. [37], another hypothesis could be that activated B cells take up more C3 from the surrounding to modulate gene expression, leading to possible inflammatory changes in CRSwNP. An intracellular role for C3 in B cell activation and homeostasis, as it was shown for T cells, has also to be considered [28]. The restricted availability of patient material and the aspect of a certain heterogeneity using individual patient samples describe the limitations of this study.

## 5. Conclusions

In summary, we have shown a C3-C3aR axis occupation in nasal polyps together with an augmented intracellular C3 repertoire in B cells which could refer to a prominent role for complement in disease progression. Understanding the exact mechanisms leading to increased C3 progression would pave the way for potential treatment options.

## Figures and Tables

**Table 1:** Patients' characteristics grouped by methodology. Y: yes; N: no; U: unknown.

	Sample size	Eosinophilic infiltrate			Asthma			Respiratory allergies		
		Y	N	U	Y	N	U	Y	N	U
Microarray	8	4	1	3	0	8	0	1	8	0
qPCR	13	6	4	3	1	12	0	3	10	0
Western blot	15	10	5	0	2	13	0	3	7	5
Immunohistochemistry	7	5	2	0	0	0	0	1	6	0
Flow cytometry	10	6	3	1	3	7	0	6	4	0
qPCR (healthy)	12	0			0			0		

**Table 2:** TaqMan™ Gene Expression Assays.

Gene symbol	Assay ID	Reference sequence	Length of amplicon
ACTB	Hs99999903_m1	NM_001101.3	171 bp
C3	Hs00163811_m1	NM_000064.2	88 bp
C3AR1	Hs00269693_s1	NM_004054.2	82 bp
C5	Hs00156197_m1	NM_001735.2	143 bp
C5AR1	Hs00704891_s1	NM_001736.3	68 bp
C5AR2	Hs01933768_s1	NM_001271749.1 NM_001271750.1 NM_018485.2	85 bp

## Data Availability

The data generated during this study are available from the corresponding author on reasonable request.

## References

[1] Hastan D., Fokkens W. J., Bachert C. (2011). Chronic rhinosinusitis in Europe--an underestimated disease. A GA^2^LEN study. *Allergy*.

[2] Larsen P. L., Tos M. (2004). Origin of nasal polyps: an endoscopic autopsy study. *The Laryngoscope.*.

[3] Van Bruaene N., Derycke L., Perez-Novo C. A. (2009). TGF-*β* signaling and collagen deposition in chronic rhinosinusitis. *Journal Allergy and Clinical Immunology*.

[4] Newton J. R., Ah-See K. W. (2008). A review of nasal polyposis. *Therapeutics and Clinical Risk Management*.

[5] Van Zele T., Claeys S., Gevaert P. (2006). Differentiation of chronic sinus diseases by measurement of inflammatory mediators. *Allergy*.

[6] STOOP A., VANDERHEIJDEN H., BIEWENGA J., VANDERBAAN S. (1993). Eosinophils in nasal polyps and nasal mucosa: an immunohistochemical study. *The Journal of Allergy and Clinical Immunology*.

[7] Boase S., Foreman A., Cleland E. (2013). The microbiome of chronic rhinosinusitis: culture, molecular diagnostics and biofilm detection. *BMC Infectious Diseases*.

[8] Fokkens W. J., Lund V. J., Mullol J. (2012). EPOS 2012: European position paper on rhinosinusitis and nasal polyps 2012. A summary for otorhinolaryngologists. *Rhinology*.

[9] Lam K., Schleimer R., Kern R. C. (2015). The etiology and pathogenesis of chronic rhinosinusitis: a review of current hypotheses. *Current Allergy and Asthma Reports*.

[10] Montone K. T. (2013). Role of fungi in the pathophysiology of chronic rhinosinusitis: an update. *Current Allergy and Asthma Reports*.

[11] Pezato R., Świerczyńska-Krępa M., Niżankowska-Mogilnicka E., Derycke L., Bachert C., Pérez-Novo C. A. (2012). Role of imbalance of eicosanoid pathways and staphylococcal superantigens in chronic rhinosinusitis. *Allergy*.

[12] Soyka M. B., Wawrzyniak P., Eiwegger T. (2012). Defective epithelial barrier in chronic rhinosinusitis: the regulation of tight junctions by IFN-*γ* and IL-4. *Journal of Allergy and Clinical Immunology*.

[13] Tantilipikorn P., Bunnag C., Nan Z., Bachert C. (2012). Staphylococcus aureus superantigens and their role in eosinophilic nasal polyp disease. *Asian Pacific Journal of Allergy and Immunology*.

[14] Orlandi R. R., Kingdom T. T., Hwang P. H. (2016). International consensus statement on allergy and rhinology: rhinosinusitis Executive Summary. *International Forum of Allergy & Rhinology*.

[15] Kolev M., Friec G. L., Kemper C. (2014). Complement -- tapping into new sites and effector systems. *Nature Reviews. Immunology*.

[16] Klos A., Tenner A. J., Johswich K.-O., Ager R. R., Reis E. S., Köhl J. (2009). The role of the anaphylatoxins in health and disease. *Molecular Immunology*.

[17] Ricklin D., Hajishengallis G., Yang K., Lambris J. D. (2010). Complement: a key system for immune surveillance and homeostasis. *Nature Immunology*.

[18] Sarma J. V., Ward P. A. (2011). The complement system. *Cell and Tissue Research*.

[19] Mulligan J. K., Patel K., Williamson T. (2018). C3a receptor antagonism as a novel therapeutic target for chronic rhinosinusitis. *Mucosal Immunology*.

[20] Schlosser R. J., Mulligan R. M., Casey S. E., Varela J. C., Harvey R. J., Atkinson C. (2010). Alterations in gene expression of complement components in chronic rhinosinusitis. *American Journal of Rhinology & Allergy*.

[21] Lane A. P., Truong-Tran Q.-A., Myers A., Bickel C., Schleimer R. P. (2018). Serum amyloid A, properdin, complement 3, and toll-like receptors are expressed locally in human sinonasal tissue. *American Journal of Rhinology*.

[22] Van Zele T., Coppieters F., Gevaert P., Holtappels G., Van Cauwenberge P., Bachert C. (2009). Local complement activation in nasal polyposis. *The Laryngoscope.*.

[23] Van Roey G. A., Vanison C. C., Wu J. (2017). Classical complement pathway activation in the nasal tissue of patients with chronic rhinosinusitis. *Journal of Allergy and Clinical Immunology*.

[24] Tan B. K., Peters A. T., Schleimer R. P., Hulse K. E. (2018). Pathogenic and protective roles of B cells and antibodies in patients with chronic rhinosinusitis. *The Journal of Allergy and Clinical Immunology*.

[25] Fleige S., Pfaffl M. W. (2006). RNA integrity and the effect on the real-time qRT-PCR performance. *Molecular Aspects of Medicine*.

[26] Könnecke M., Böscke R., Waldmann A. (2014). Immune imbalance in nasal polyps of Caucasian chronic rhinosinusitis patients is associated with a downregulation of E-selectin. *Journal of Immunology Research*.

[27] DiScipio R. G., Daffern P. J., Jagels M. A., Broide D. H., Sriramarao P. (1999). A comparison of C3a and C5a-mediated stable adhesion of rolling eosinophils in postcapillary venules and transendothelial migration in vitro and in vivo. *Journal of Immunology Baltimore M,d: 1950*.

[28] Liszewski M. . K., Kolev M., Friec G. L. (2013). Intracellular complement activation sustains T cell homeostasis and mediates effector differentiation. *Immunity*.

[29] DeZern A. E., Brodsky R. A. (2015). Paroxysmal nocturnal hemoglobinuria: a complement-mediated hemolytic anemia. *Hematology/Oncology Clinics of North America*.

[30] Kavanagh D., Goodship T. H., Richards A. (2013). Atypical hemolytic uremic syndrome. *Seminars in Nephrology*.

[31] Hulse K. E., Stevens W. W., Tan B. K., Schleimer R. P. (2015). Pathogenesis of nasal polyposis. *Clinical & Experimental Allergy*.

[32] Drouin S. M., Corry D. B., Hollman T. J., Kildsgaard J., Wetsel R. A. (2002). Absence of the complement anaphylatoxin C3a receptor suppresses Th2 effector functions in a murine model of pulmonary allergy. *The Journal of Immunology*.

[33] Kulkarni H. S., Liszewski M. K., Brody S. L., Atkinson J. P. (2018). The complement system in the airway epithelium: an overlooked host defense mechanism and therapeutic target?. *Journal of Allergy and Clinical Immunology*.

[34] Peng Q., Li K., Patel H., Sacks S. H., Zhou W. (2006). Dendritic cell synthesis of C3 is required for full T cell activation and development of a Th1 phenotype. *The Journal of Immunology*.

[35] Lubbers R., van Essen M. F., van Kooten C., Trouw L. A. (2017). Production of complement components by cells of the immune system. *Clinical and Experimental Immunology*.

[36] Elvington M., Liszewski M. K., Bertram P., Kulkarni H. S., Atkinson J. P. (2017). A C3(H20) recycling pathway is a component of the intracellular complement system. *The Journal of Clinical Investigation*.

[37] Kremlitzka M., Nowacka A. A., Mohlin F. C., Bompada P., De Marinis Y., Blom A. M. (2019). Interaction of serum-derived and internalized C3 with DNA in human B cells-a potential involvement in regulation of gene transcription. *Frontiers in Immunology*.

[38] Thompson C. B., Scher I., Schaefer M. E., Lindsten T., Finkelman F. D., Mond J. J. (1984). Size-dependent B lymphocyte subpopulations: relationship of cell volume to surface phenotype, cell cycle, proliferative response, and requirements for antibody production to TNP-Ficoll and TNP-BA. *J Immunol Baltim Md 1950*.

[39] Feldman S., Kasjanski R., Poposki J. (2017). Chronic airway inflammation provides a unique environment for B cell activation and antibody production. *Clinical & Experimental Allergy*.

[40] Hulse K. E., Norton J. E., Suh L. (2013). Chronic rhinosinusitis with nasal polyps is characterized by B-cell inflammation and EBV-induced protein 2 expression. *Journal of Allergy and Clinical Immunology*.

[41] Gevaert P., Nouri-Aria K. T., Wu H. (2013). Local receptor revision and class switching to IgE in chronic rhinosinusitis with nasal polyps. *Allergy*.

[42] Kato A., Peters A., Suh L. (2008). Evidence of a role for B cell-activating factor of the TNF family in the pathogenesis of chronic rhinosinusitis with nasal polyps. *Journal of Allergy and Clinical Immunology*.

[43] Tan B. K., Li Q.-Z., Suh L. (2011). Evidence for intranasal antinuclear autoantibodies in patients with chronic rhinosinusitis with nasal polyps. *Journal of Allergy and Clinical Immunology*.

[44] Van Zele T., Gevaert P., Holtappels G., van Cauwenberge P., Bachert C. (2007). Local immunoglobulin production in nasal polyposis is modulated by superantigens. *Clinical & Experimental Allergy*.

